# Supplementary material for: Inhibiting the Interaction Between Phospholipase A2 and Phospholipid Serine as a Potential Therapeutic Method for Pneumonia
Source: Curr Issues Mol Biol. 2025 Jul 4;47(7):516. doi: 10.3390/cimb47070516 (PMC12293276; doi:10.3390/cimb47070516)
Supplement: Supplementary file 1 [file cimb-47-00516-s001.zip › cimb-3637644-supplementary.pdf]

Supplementary information

Inhibiting the Interaction Between Phospholipase A2 and Phospholipid Serine as a Potential Therapeutic Method for Pneumonia

Jianyu Wang, Huanchun Xing, Lin Wang, Zhongxing Xu, Xin Sui, Yuan Luo, Jun Yang * and Yongan Wang *

State Key Laboratory of Toxicology and Medical Countermeasures, Academy of Military Medical Sciences, Beijing, 100850, China

* Correspondence: ajaway@126.com (J.Y.); yonganw@126.com (Y.W.)

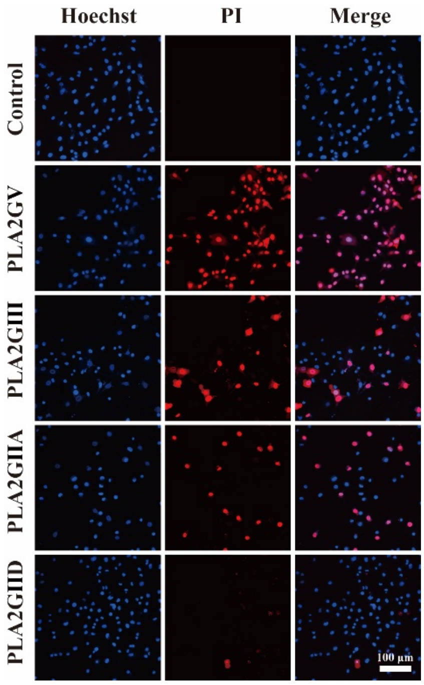

Figure S1. Confocal microscopy was used to observe the damage of A549 cells caused by the mixture of different subtypes of PLA2 and PS.

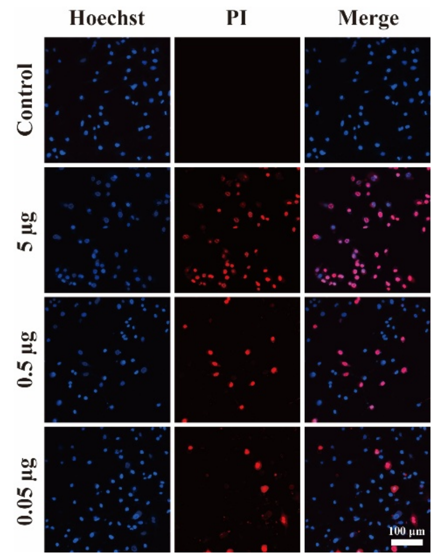

Figure S2. Confocal microscopy was used to observe the damage of A549 cells caused by different concentrations of lyso-PLs.

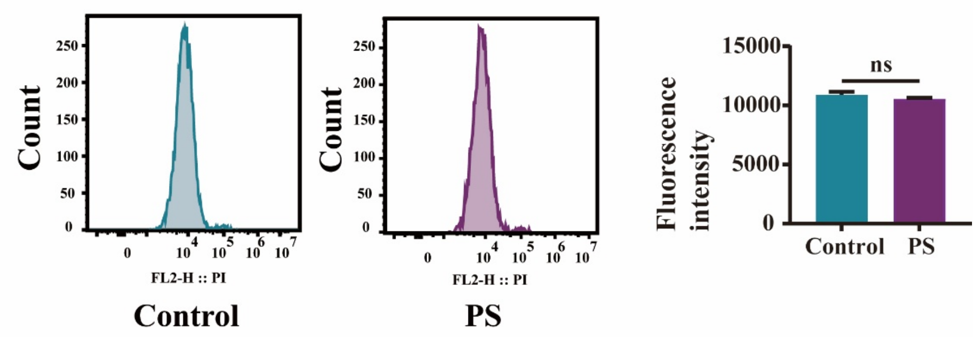

Figure S3. The results of flow cytometry analysis showing staining of RAW264.7 cells with PI.

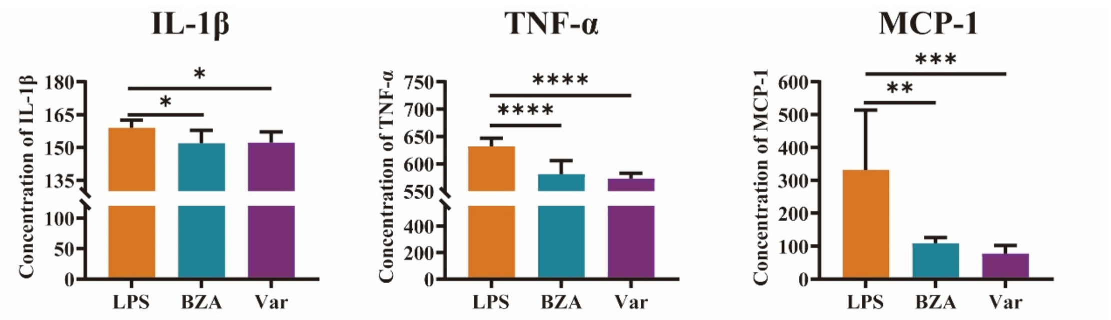

Figure S4. ELISA-measured levels of IL-1β, TNF-α, and MCP-1 in the lung tissue after PLA2 inhibitor therapy for pneumonia.

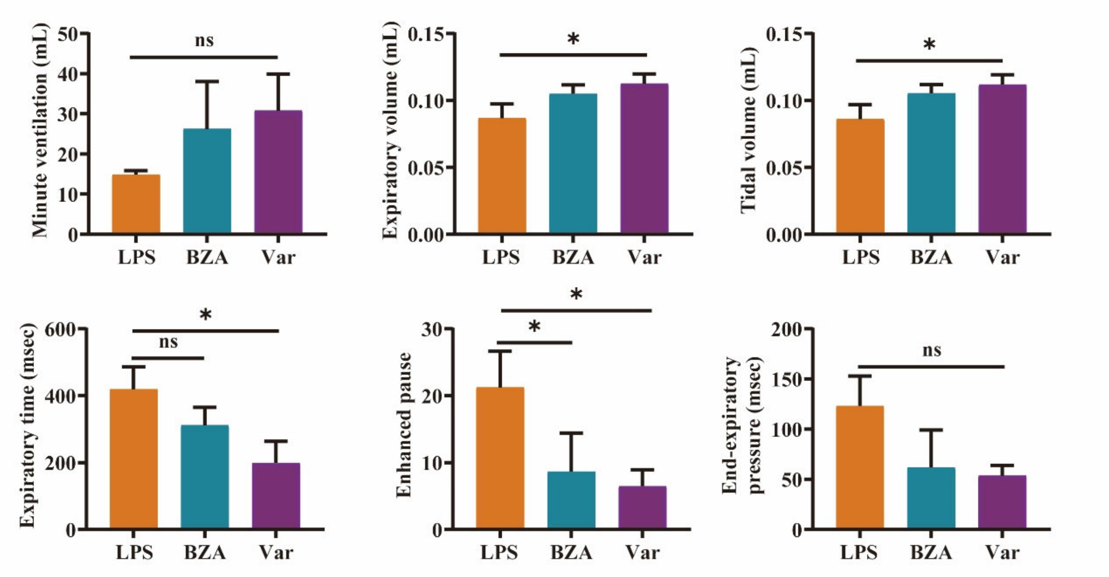

Figure S5. Pulmonary function results following PLA2 inhibitor therapy for pneumonia.
